# Supplementary material for: Viscoelasticity of diverse biological samples quantified by Acoustic Force Microrheology (AFMR)
Source: Commun Biol. 2024 Jun 4;7:683. doi: 10.1038/s42003-024-06367-3 (PMC11150513; doi:10.1038/s42003-024-06367-3)
Supplement: Supplementary file 2 — Supplementary Information [file 42003_2024_6367_MOESM2_ESM.pdf]

SUPPLEMENTARY INFORMATION

Viscoelasticity of diverse biological samples quantified by Acoustic Force Microrheology (AFMR)

Giulia Bergamaschi[†1], Kees-Karel H. Taris[†1], Andreas S. Biebricher[1],
Xamanie M.R. Seymonson[1], Hannes Witt[1], Erwin J.G. Peterman[1], Gijs J.L. Wuite[1*]

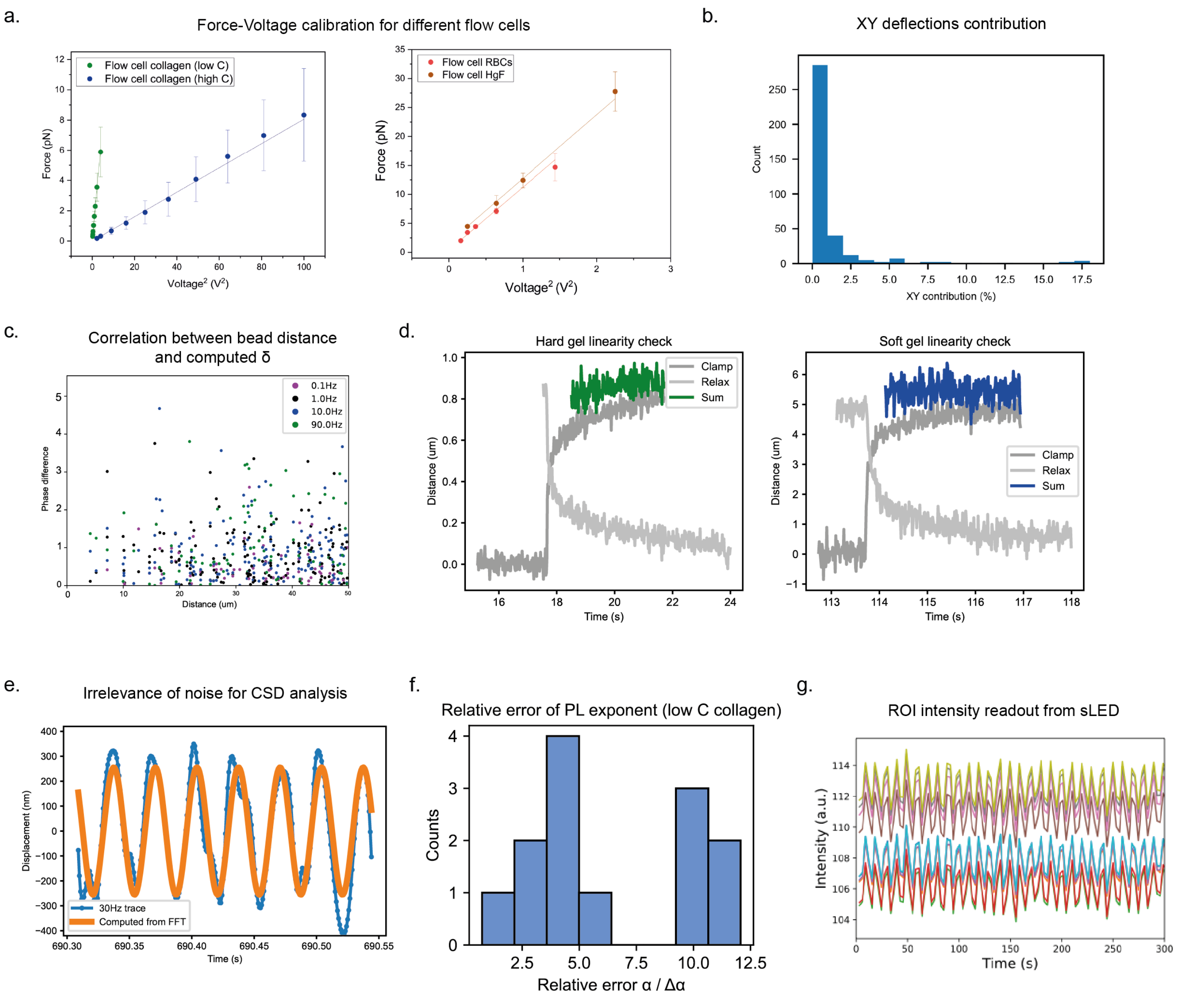

Supplementary Fig. 1: Characterisation of AFMR setup and collagen measurements. a) *Force calibration procedure for different flow cells and beads. Plotted are the measured forces with SEM (as deduced using the shooting-up calibration procedure) vs the input voltage squared. (I) for RBCs and HgF experiments, and (II) for collagen experiments. The data is averaged over many 'shooting' beads in the FOV (N_{RBCs} = 15, N_{HgF} =26, N_{lowC} = 96, N_{highC} = 102), retrieving the conversion factors:* $ß_{RBCs}$ = 12.1 ± 0.5, $ß_{HgF}$ = 12.5 ± 0.2, $ß_{lowC}$ = 0.08 ± 0.01, $ß_{highC}$ = 1.7 ± 0.1. *The measurement over different voltages is important since it verifies that the force depends on the voltage-squared, as the theory of the Stokes drag in a Newtonian fluid stipulates. The lines show the corresponding linear equation fit curves from which then the conversion factor a is directly deduced. Note that the slope (i.e., the conversion factor) is different for each sample, which is not only caused by the use of different bead sizes, but by the fact that the strength of the acoustic field (and thus the conversion factor, see equation 1) can vary between different flow cells,* ***b)*** *Beads are predominantly displaced in the Z-direction. The relative contribution of XY displacements to the total (XYZ) displacement for the beads measured in high collagen concentration (Fig. 3b) shows that beads are mostly displaced in the Z-direction by application of a force. In only ~7,5% of the cases beads showed a relative displacement contribution > 5% in XY-direction (6 out of 80 beads). Note that this plot counts each frequency for the same bead as a separate event, therefore the total count exceeds 80.* ***c)*** *A plot of the phase difference between bead pairs vs. their distance for different frequencies does not give any indication that measured phase values show significant correlation with the inter-bead distance at distances >5 µm,* ***d)*** *High-frequency noise observed for the case of low collagen concentration does not interfere with proper data fitting. Overlay of the fitting function (orange) and the raw data (blue) shows that the FFT analysis faithfully follows the raw data. Furthermore, this larger noise at high frequencies is nearly compensated by the greatly increased number of oscillations (50 repetitions at 30 Hz) which accounts for the fact that the relative error at higher frequencies is not substantially larger (Fig. 2d).* ***e)*** *Linearity check for two collagen samples used. This can be deduced from the fact that the sum of overlayed clamp and relaxation curves is constant, i.e. the material recovers completely after stress is applied* [1,2]*.* ***f)*** *The distribution of relative SD-values for calculated PL exponents deduced from repeated single-bead measurements over time (Fig. 2cd; n=181; N=2) yields a mean RE of 6%.* ***g)*** *Accuracy of sLED read-out. Plots of intensity over time for the sLED intensity as detected by the CMOS camera for a 100 Hz oscillation. Each color represents the intensity readout for a different ROI. The fact that all the*

traces show virtually identical oscillation characteristics - just with a different off-set verifies that the force oscillation can be accurately followed by tracking the sLED intensity.

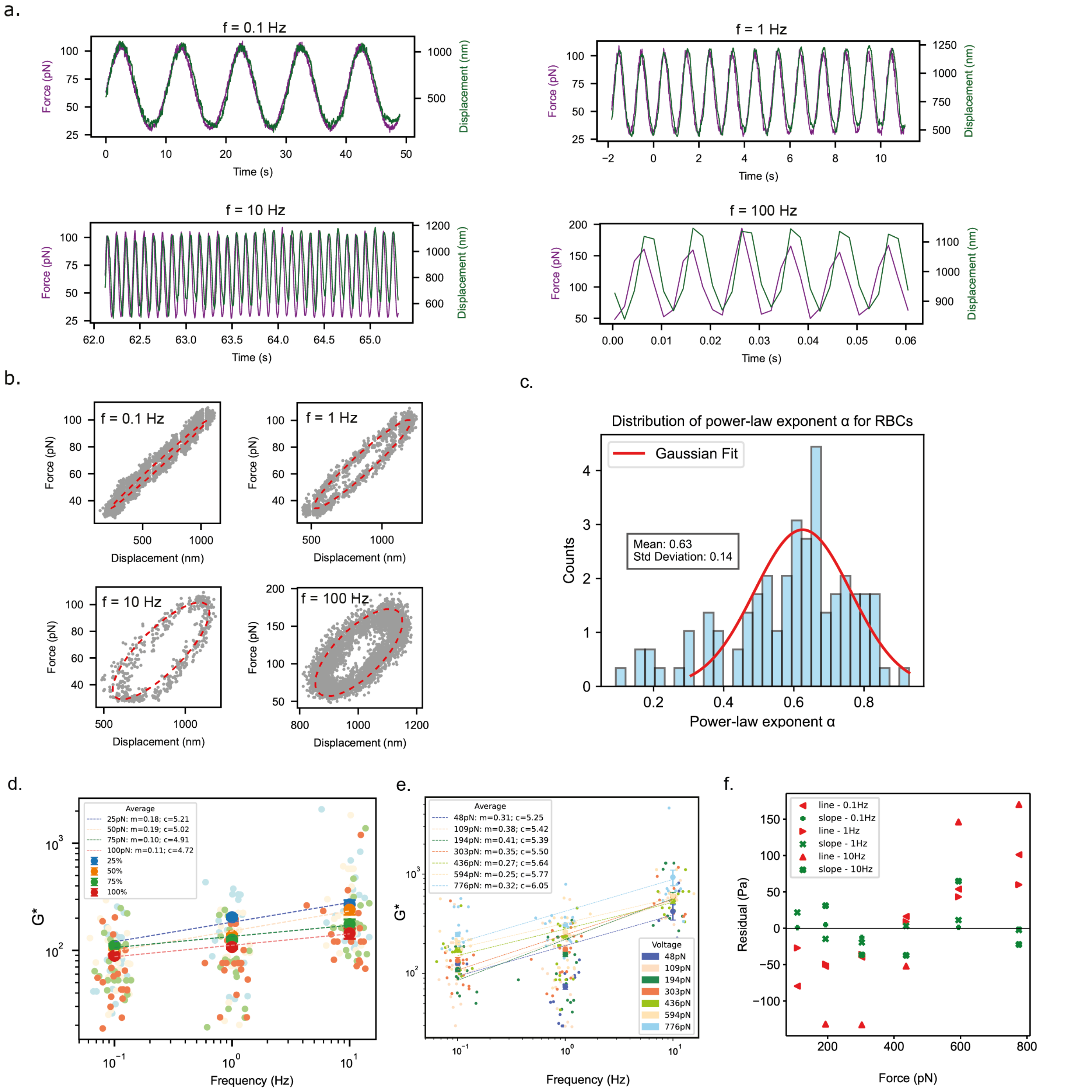

Supplementary Fig. 2: RBC raw AFMR data and individual measurements/fits for different force settings. a) *Typical raw traces of oscillating 6.59 µm silica beads on top of red blood cells. Force (magenta) and distance (green) versus time plots at modulation frequencies of 0.1Hz, 1Hz, 10Hz, and 100Hz.* ***b)*** *Force-displacement (Lissajous) plots for the raw traces shown in a). Similar to the collagen gel results (Fig. 2a), the phase delay increases significantly with the frequency. The Lissajous plots can be very well fitted by an ellipse indicates that we are probing the linear stress regime.* ***c)*** *Distribution of power-law exponent α for RBCs (n=139; N=2) computed by fitting single-cell G'' data at f>1Hz shows a RE~21%.* ***d)*** *The effect of pre-stress (n=17-24; N=2-3) and* ***e)*** *modulation depth (n=10-19; N=4-6) of the force on the complex modulus G*. The average values (and their trend lines) of the different pre-stresses or depths show no consistent effect on the power law exponent. These findings match those of [3], which also reported no relevant change of the exponent with pre-stress.* ***f)*** *Residuals after fitting either a line (y = a) or a slope (y = ax + b). The pattern shows that a slope better fits the data, indicating stiffening at increasing forces.*

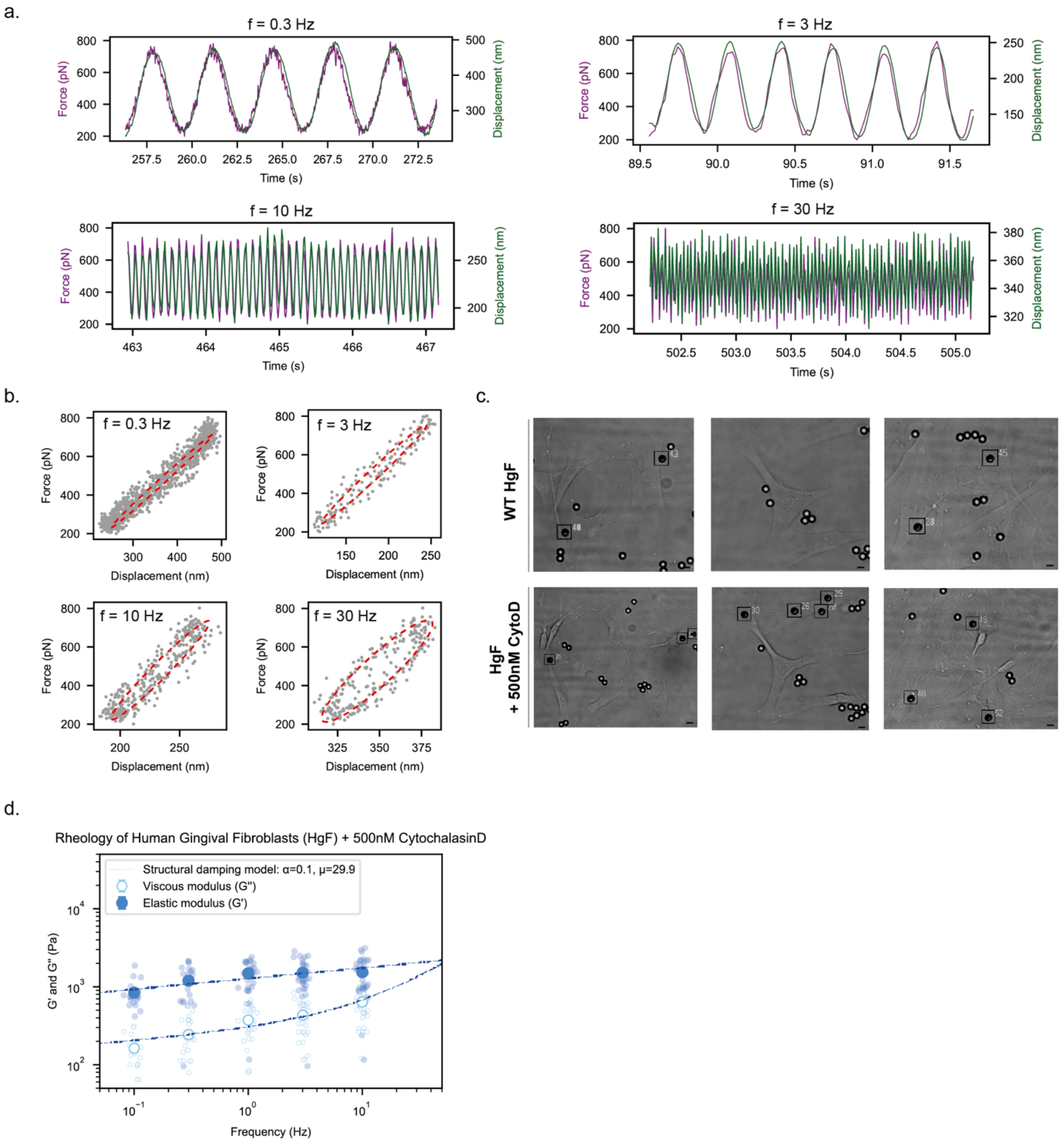

***Supplementary Fig. 3: HgF raw AFMR data**. **a)** Typical raw traces of oscillating 6.59 µm silica beads on top of HgF. Force (magenta) and distance (green) versus time plots at modulation frequencies of 0.3Hz, 3Hz, 10Hz, and 30Hz **b)** Force-displacement (Lissajous) plots for the raw traces shown in (a). Similar to the RBC data (Fig. S2B), the elliptical shape of the Lissjous plots demonstrates that we probe the linear regime. **c)** Zoomed-in wide-field images before (top) and after treatment (bottom) of human gingival fibroblasts (HgF) with cytoD. As is well documented [4,5], cells respond to the treatment by producing thinner filopodia and displaying a clumped-up cell body, indicating that the measured change in the viscoelastic properties reflects the successful drug treatment of the cell. Note that the position of the beads might change after treatment, due to the flushing procedure and the cells motility. Top and bottom images show the same cells before and after treatment. Scale bars = 20µm. **d)** AFMR measurements of HgF after addition of 500nM cytochalasinD. Single bead G'-G'' data is shown, while larger marker is the mean.*

a.

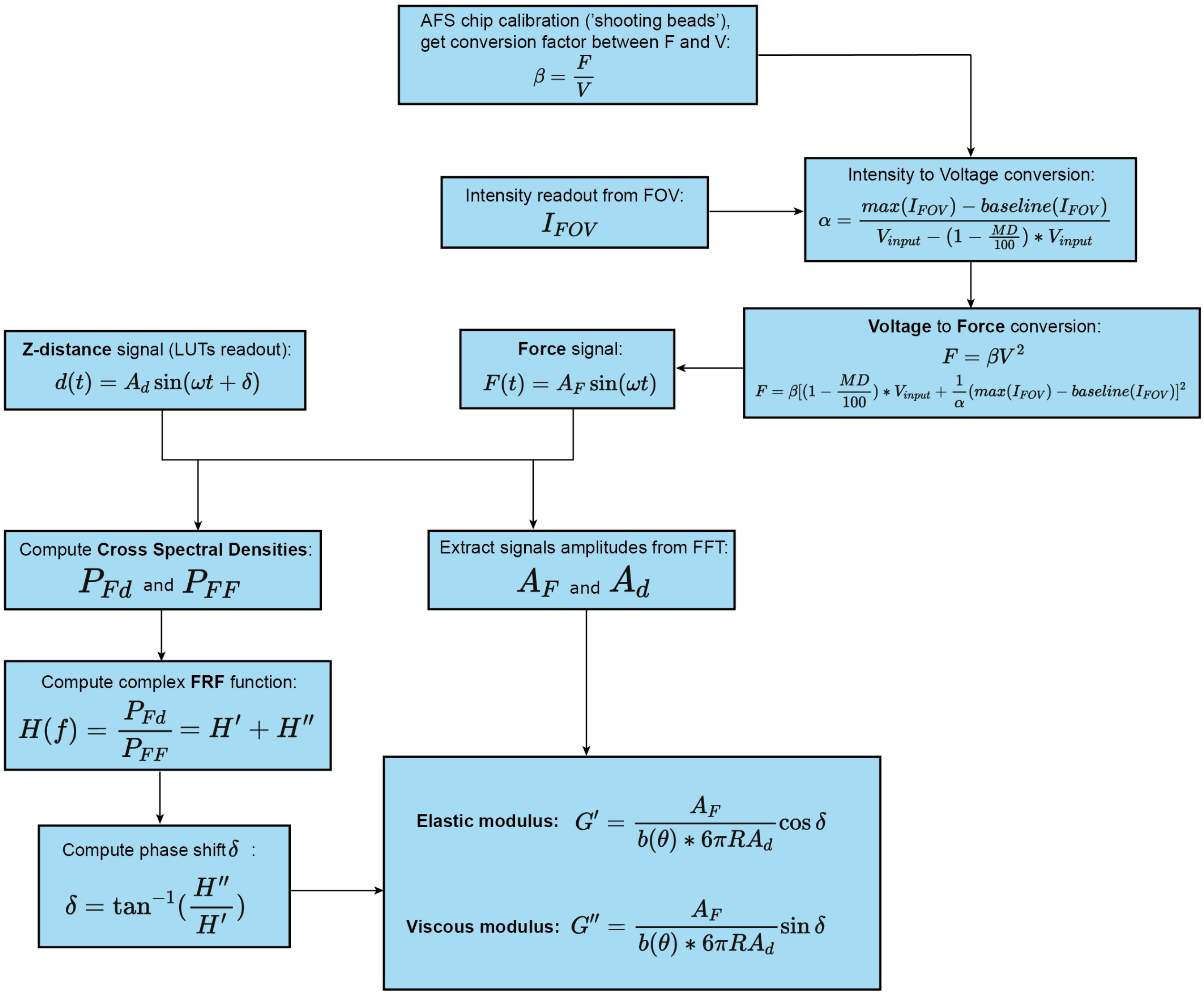

b.

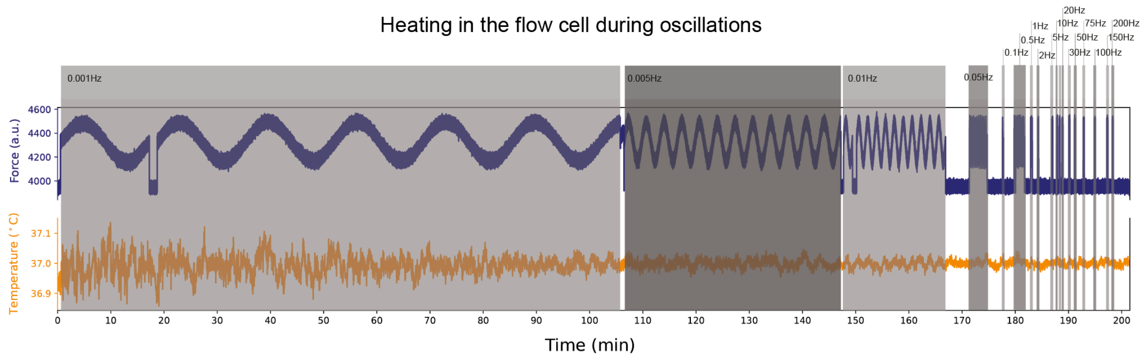

Supplementary Fig. 4: Analysis pipeline and temperature control during measurements. a) *The analysis pipeline of the python script performing FRF analysis as described in the STAR Methods – Data analysis workflow.* ***b)*** *The temperature fluctuations (orange) during different modulation frequencies (blue, 0.001-200Hz) - each in a different grey box - remain below 0.15 °C. Thus, we conclude that force oscillations do not influence the temperature of the flow cell when kept at 37 °C.*

Supplementary Table 1: Comparison of relative G'-G'' errors.

	Collagen		Red Blood cells (RBCs)	
**Frequency (Hz)**	**RE(G')**	**RE(G'')**	**RE(G')**	**RE(G'')**
0.03	12.2	20.72	/	/
0.1	11.74	20.97	12.39	53.82
0.3	13.74	17.69	10.72	19.48
1	16.78	15.02	11.13	23.08
3	14.05	10.5	12.34	13.34
10	12.32	10.74	6.29	3.51
30	17.53	14.37	7.11	7.80
100	24.89	17.09	/	/

***Supplementary Table 1**: **Comparison of relative G'-G'' errors for collagen and RBCs.** The relative errors are computed for all beads in a single FOV, repeatedly measured over time.*

References

1. Findley, W. N., Lai, J. S., Onaran, K. & Christensen, R. M. *Creep and Relaxation of Nonlinear Viscoelastic Materials With an Introduction to Linear Viscoelasticity*. *Journal of Applied Mechanics* vol. 44 (1977).
2. Sorkin, R. *et al.* Probing cellular mechanics with acoustic force spectroscopy. *Mol Biol Cell* **29**, (2018).
3. Yoon, Y. Z., Kotar, J., Brown, A. T. & Cicuta, P. Red blood cell dynamics: From spontaneous fluctuations to non-linear response. *Soft Matter* **7**, 2042–2051 (2011).
4. Rotsch, C. & Radmacher, M. Drug-induced changes of cytoskeletal structure and mechanics in fibroblasts: An atomic force microscopy study. *Biophys J* **78**, 520–535 (2000).
5. Ayala, Y. A. *et al.* Effects of cytoskeletal drugs on actin cortex elasticity. *Exp Cell Res* **351**, 173–181 (2017).
