# Supplementary material for: Viscoelasticity of diverse biological samples quantified by Acoustic Force Microrheology (AFMR)
Source: Commun Biol. 2024 Jun 4;7:683. doi: 10.1038/s42003-024-06367-3 (PMC11150513; doi:10.1038/s42003-024-06367-3)
Supplement: Supplementary file 3 — Description of Additional Supplementary Files [file 42003_2024_6367_MOESM3_ESM.pdf]

Description of Additional Supplementary Files

File name: Supplementary Data 1

Description: The source data behind the graphs in the paper, each sheet is a different subfigure (ie 2C, 2D etc).

File name: Supplementary Data 2

Description: the top x-axis (time) from Supplementary Figure 4B

File name: Supplementary Data 3

Description: the force values for Supplementary Figure 4B
