# Supplementary material for: Viscoelasticity of diverse biological samples quantified by Acoustic Force Microrheology (AFMR)
Source: Commun Biol. 2024 Jun 4;7:683. doi: 10.1038/s42003-024-06367-3 (PMC11150513; doi:10.1038/s42003-024-06367-3)
Supplement: Supplementary file 7 — Reporting summary [file 42003_2024_6367_MOESM7_ESM.pdf]

nature portfolio

Corresponding author(s): G. Bergamaschi
G.J.L. Wuite

Last updated by author(s): 26/03/2024

Reporting Summary

Nature Portfolio wishes to improve the reproducibility of the work that we publish. This form provides structure for consistency and transparency in reporting. For further information on Nature Portfolio policies, see our Editorial Policies and the Editorial Policy Checklist.

Statistics

For all statistical analyses, confirm that the following items are present in the figure legend, table legend, main text, or Methods section.

n/a	Confirmed	
☐	☒	The exact sample size (*n*) for each experimental group/condition, given as a discrete number and unit of measurement
☐	☒	A statement on whether measurements were taken from distinct samples or whether the same sample was measured repeatedly
☐	☒	The statistical test(s) used AND whether they are one- or two-sided<br>*Only common tests should be described solely by name; describe more complex techniques in the Methods section.*
☒	☐	A description of all covariates tested
☒	☐	A description of any assumptions or corrections, such as tests of normality and adjustment for multiple comparisons
☐	☒	A full description of the statistical parameters including central tendency (e.g. means) or other basic estimates (e.g. regression coefficient) AND variation (e.g. standard deviation) or associated estimates of uncertainty (e.g. confidence intervals)
☒	☐	For null hypothesis testing, the test statistic (e.g. *F*, *t*, *r*) with confidence intervals, effect sizes, degrees of freedom and *P* value noted<br>*Give P values as exact values whenever suitable.*
☒	☐	For Bayesian analysis, information on the choice of priors and Markov chain Monte Carlo settings
☒	☐	For hierarchical and complex designs, identification of the appropriate level for tests and full reporting of outcomes
☒	☐	Estimates of effect sizes (e.g. Cohen's *d*, Pearson's *r*), indicating how they were calculated

Our web collection on statistics for biologists contains articles on many of the points above.

Software and code

Policy information about availability of computer code

Data collection	The AFS setup runs on LabView 2016 for data collection (LUTs readout and LED intensity readout)
Data analysis	For data analysis, we made custom MR analysis pipelines using Python 3.8.8. All codes and test data are available on Github: https://github.com/PLSysGitHub/AFMRv23

For manuscripts utilizing custom algorithms or software that are central to the research but not yet described in published literature, software must be made available to editors and reviewers. We strongly encourage code deposition in a community repository (e.g. GitHub). See the Nature Portfolio guidelines for submitting code & software for further information.

Data

Policy information about availability of data

All manuscripts must include a data availability statement. This statement should provide the following information, where applicable:

- Accession codes, unique identifiers, or web links for publicly available datasets
- A description of any restrictions on data availability
- For clinical datasets or third party data, please ensure that the statement adheres to our policy

A selection of datasets is already available on Github, as well as on dataverse (https://dataverse.nl/privateurl.xhtml?token=9e0a9601-ccce-428a-920e-fa0b0484fa40). Full datasets will be published on dataverse prior publication.

Research involving human participants, their data, or biological material

Policy information about studies with human participants or human data. See also policy information about sex, gender (identity/presentation), and sexual orientation and race, ethnicity and racism.

Reporting on sex and gender	We report that the Human Gingival Fibroblasts were extracted from the residual gingiva on wisdom tooth of a healthy 20 years old F (sex). We also included sex and age of volunteers who donated RBCs by finger pricking.
Reporting on race, ethnicity, or other socially relevant groupings	These variables do not apply to the studies conducted here.
Population characteristics	For the RBCs study, the age of the participants was between 25 and 30. Sex was mixed. Human Gingival Fibroblasts were donated from the group of T. De Vries (ACTA, Amsterdam), and extracted from the residual gingiva on wisdom tooth of a healthy 20 years old female.
Recruitment	Volunteers for RBCs finger pricking were selected among group members.
Ethics oversight	Vrije Universiteit Amsterdam and VUmc

Note that full information on the approval of the study protocol must also be provided in the manuscript.

Field-specific reporting

Please select the one below that is the best fit for your research. If you are not sure, read the appropriate sections before making your selection.

☒ Life sciences ☐ Behavioural & social sciences ☐ Ecological, evolutionary & environmental sciences

For a reference copy of the document with all sections, see nature.com/documents/nr-reporting-summary-flat.pdf

Life sciences study design

All studies must disclose on these points even when the disclosure is negative.

Sample size	As AFS provides highly-parallel measurements, we were able to measure N > 50 beads for each experimental condition. Upon data exclusion (se below) the final statistics were as follows (as reported in Methods): 1) collagen N~130-180, 2) RBCS N~143, 3) HgF N~70. This provides enough statistics to extract average moduli from the populations, but also allows us to extract multiple single-bead information to identify local heterogeneities in force response.
Data exclusions	Data exclusion is discussed in detail in the Methods; the raw recorded traces were observed individually and filtered out if:<br>- Showed error in frequency response function analysis, i.e. incorrect frequency peak due to noisy trace (position of the peak not matching input frequency);<br>- Showed errors in tracking (due to interferences in LUTs);<br>- Showed too small/noisy amplitude (< 20 nm, which is the z-resolution of the instrument on these experiments 41);<br>- Showed strong non-linearities in the Lissajous plots;<br>- Showed ruptures or other inconsistencies in the traces, such as slipping. These features are directly identifiable from the raw traces, as in both cases the 'ruptured/slipped bead would then move towards the node, resulting in a linear 'shooting-up' trajectory, z > 10µm.
Replication	Firstly, experiments were repeated on different experimental days (N > 2) to ensure reproducibility and avoid day-to-day bias. Furthermore, 'measurements reproducibility' is a core theme of the paper, as we present here a quantification of the instrument precision/error and reproducibility by re-measuring the same beads (both collagen-embedded and on top of cells) over time for t > 1 hour.
Randomization	Healthy volunteers for finger pricking were selected among group members, irrespective of sex or age. Concerning human gingival fibroblasts, measurements were performed strictly on cells between passages 6-8, as older cells tended to be much stiffer due to age.
Blinding	Investigators were not blind to the sex and age of participants for the RBCs study, but this has no influence on the data collection or results. Human gingival fibroblast were a kind gift of T. de Vries (ACTA, Amsterdam) and were collected from an anonymous healthy volunteer (F).

Reporting for specific materials, systems and methods

We require information from authors about some types of materials, experimental systems and methods used in many studies. Here, indicate whether each material, system or method listed is relevant to your study. If you are not sure if a list item applies to your research, read the appropriate section before selecting a response.

Materials & experimental systems

n/a	Involved in the study
☒	☐ Antibodies
☐	☒ Eukaryotic cell lines
☒	☐ Palaeontology and archaeology
☒	☐ Animals and other organisms
☒	☐ Clinical data
☒	☐ Dual use research of concern
☒	☐ Plants

Methods

n/a	Involved in the study
☒	☐ ChIP-seq
☒	☐ Flow cytometry
☒	☐ MRI-based neuroimaging

Eukaryotic cell lines

Policy information about cell lines and Sex and Gender in Research

Cell line source(s)

Human Gingival Fibroblasts were donated from the group of T. De Vries (ACTA, Amsterdam), and extracted from the residual gingiva on wisdom tooth of a healthy 20 years old female. Sampling from the donors was conducted at VU University Hospital (Vrije Universiteit, Amsterdam, The Netherlands). All the individuals signed informed consent and samples were coded to guarantee the anonymity of the donors as required by Dutch law. Researchers handling the fibroblasts could not retrieve the identity of the donors.

Authentication

Cell line HgF has been authenticated via standard protocol at VU University Hospital (Vrije Universiteit, Amsterdam, The Netherlands)

Mycoplasma contamination

Cell lines tested negative for mycoplasma.

Commonly misidentified lines
(See ICLAC register)

Name any commonly misidentified cell lines used in the study and provide a rationale for their use.

Plants

Seed stocks

Report on the source of all seed stocks or other plant material used. If applicable, state the seed stock centre and catalogue number. If plant specimens were collected from the field, describe the collection location, date and sampling procedures.

Novel plant genotypes

Describe the methods by which all novel plant genotypes were produced. This includes those generated by transgenic approaches, gene editing, chemical/radiation-based mutagenesis and hybridization. For transgenic lines, describe the transformation method, the number of independent lines analyzed and the generation upon which experiments were performed. For gene-edited lines, describe the editor used, the endogenous sequence targeted for editing, the targeting guide RNA sequence (if applicable) and how the editor was applied.

Authentication

Describe any authentication procedures for each seed stock used or novel genotype generated. Describe any experiments used to assess the effect of a mutation and, where applicable, how potential secondary effects (e.g. second site T-DNA insertions, mosiacism, off-target gene editing) were examined.
